# Substance Use in Mild-COVID-19 Patients: A Retrospective Study

**DOI:** 10.3389/fpubh.2021.634396

**Published:** 2021-03-04

**Authors:** Flavia Ismael, Beatriz Zaramella, Tatiane Battagin, João C. S. Bizario, Júlia Gallego, Victoria Villela, Lilian Bezerra de Queiroz, Fabio E. Leal, Julio Torales, Antonio Ventriglio, Megan E. Marziali, Priscila D. Gonçalves, Silvia S. Martins, João M. Castaldelli-Maia

**Affiliations:** ^1^Universidade Municipal de São Caetano do Sul, São Caetano do Sul, Brazil; ^2^ABC Center for Mental Health Studies, Santo André, Brazil; ^3^Faculdade de Medicina de Olinda, Olinda, Brazil; ^4^Department of Psychiatry, School of Medical Sciences, National University of Asunción, Asunción, Paraguay; ^5^Department of Clinical and Experimental Medicine, University of Foggia, Foggia, Italy; ^6^Department of Epidemiology, Mailman School of Public Health, Columbia University, New York, NY, United States

**Keywords:** COVID-19, alcohol, analgesics, cannabis, tobacco, benzodaizepine

## Abstract

**Background:** There is a need for prospective studies investigating substance use variations in mild COVID-19 patients. These individuals represent the majority of patients affected by the disease and are routinely treated at home, facing periods of quarantine.

**Methods:** This was a retrospective cohort study. All people who tested positive for COVID-19 and classified as mild cases (i.e., no alarm sign/symptom, no need for in-person consultation) during the treatment in the public health system of a Brazilian city with around 160,000 inhabitants were monitored by phone for all the COVID-19 symptoms listed by the Centers for Disease Control and Prevention (CDC) during the active phase of the disease (i.e., no longer experiencing symptoms, up to 14 days in mild cases). After this phase (median = 108 days after intake, IQR = 76–137), we asked these patients who were classified as experiencing mild COVID-19 (*n* = 993) about last-month substance use in three time-points: pre-COVID, just after COVID-19 acute phase (post-COVID acute phase) and in the period before survey (post-COVID follow-up phase).

**Results:** The number of COVID-19 symptoms was not associated with pre- or post-infection substance use. Pre-COVID alcohol and non-medical benzodiazepine use were associated with specific COVID-19 symptoms. However, sensitivity analyses showed that such associations could be explained by previous psychiatric and medical profiles. Alcohol and tobacco use decreased and non-medical analgesics increased in the post-COVID acute phase. However, just alcohol use remained lower in the post-COVID follow-up period. Higher pre-COVID levels of tobacco and alcohol were associated with post-COVID follow-up cannabis and non-medical analgesic use, respectively. Non-medical benzodiazepine use had positive and negative bi-directional associations with cannabis and non-medical analgesic use, respectively.

**Conclusion:** We were not able to find specific associations between substance use and COVID-19 symptomatology in the present study. Patients with mild COVID-19 should be monitored for substance use in the post-COVID-19 period, and preventive interventions for non-medical analgesic use should be implemented. Focused preventive interventions increasing the perceived risks of cannabis and non-medical benzodiazepine and analgesic use among people experiencing mild COVID-19 that reported previous substance use could be useful.

## Introduction

There is a risk for collision of two epidemics: COVID-19 and substance use (1–3). The COVID-19 pandemic is an unprecedented public health challenge, with potential for secondary effects on substance use outcomes (4). Alcohol, tobacco, and drug use have been among the top global risk factors for attributable mortality, years of life lost, years of life lived with disability, and disability-adjusted life-years in the last decades (5). People who use substances may be particularly vulnerable to COVID-19 (3, 6). There remains uncertainty, especially among those with mild COVID-19, who are the vast majority of COVID-19 patients (7).

Previous studies demonstrate that alcohol use may significantly increase the risk of contracting bacterial and viral lung infections, which could apply to SARS-CoV-2 (6). Chronic alcohol intake impairs various immunity components, such as reinforcing the inflammatory reaction and activating the CD8 response, increasing the influenzae risk infection (8). Tobacco smoking is another known risk factor for respiratory infections and functions to increase disease severity (9). However, there are mixed findings regarding the role of tobacco on the COVID-19 pandemic: a meta-analysis conducted by Patanavanich and Glantz (10) found that smokers are more likely to develop severe disease with COVID-19 compared to non-smokers, whereas another meta-analysis identified smoking as protective for COVID-19 infection (11). A recent electronic health record study showed that individuals with substance use disorder, especially those with opioid use disorder, have an increased risk for COVID-19 and its adverse outcomes (12). There is a need to further investigate the role of each substance in regards to COVID-19 clinical outcomes.

General population studies show that substance use has been predominantly increasing during the pandemic (13–18). In a web-survey during the social distance measures in Belgium, individuals reported more alcohol and tobacco use than before the lockdown (17). An extensive web survey in France also found an increase in alcohol (24.8%), tobacco (35.6%), and cannabis (31.2%) during the early phase of COVID-19 containment (16). Callinan et al. (14) conducted a cross-sectional study with 1,684 adult Australians who drink at least monthly. They found that harmful drinking decreased during social distancing measures, especially among (13, 14). In a cross-sectional survey of 12,328 adults within the 33 of Latin American and Caribbean, there was a decrease in alcohol use but a stability in heavy episodic drinking between 2019 and 2020 (during the pandemic) (18). In the U.S., alcohol use and heavy drinking before and during the COVID-19 pandemic increased by 14% in comparison to 2019 (15).

This increase has particularly affected some subgroups, such as people with previous substance use disorders, with increased levels of stress, or who engage in self-isolation (13, 14, 19, 20). In Australia, those experiencing high levels of stress have a higher increase in harmful drinking than those reporting lower stress levels (13, 14). Kim et al. (20) conducted a cross-sectional telephone survey of patients with pre-existing alcohol disorders registered in an alcohol care service in the United Kingdom, 2 months after the beginning of the containment measures. Approximately 17% had relapsed during this period. Regarding cannabis, a small survey reported an increase of 20% of cannabis use among those who engaged in self-isolation compared to those who did not. (19). It would be essential to investigate if patients with mild COVID-19 also have increased substance use after the disease's active period, as these patients could experience longer and more restrictive quarantines than the general population (7). There is a need for studies investigating substance use variations in mild COVID-19 patients.

The present retrospective cohort study aimed to investigate: differences between pre- and post-COVID-19 substance use; whether pre-COVID-19 substance use could be associated with COVID-19 amount and types of symptoms; if the number of COVID-19 symptoms would be related to post-COVID-19 substance use; and associations between pre- and post-COVID-19 substance use.

## Materials and Methods

### Ethical Approval

The present study was approved by the local ethics committee (*Comissão de Ética para Análise de Projeto de Pesquisa*- CAPPesq, protocol No. 37265620.0.0000.5510, approved on September 2nd, 2020).

### Study Design

All people who tested positive for COVID-19 and were classified as mild cases (i.e., no alarm sign/symptom, no need for in-person consultation) (21) were considered for inclusion. Participants were from a Brazilian city with around 160,000 inhabitants and were identified for inclusion during COVID-19 treatment. They were then monitored by phone for all the COVID-19 symptoms listed by the Centers for Disease Control and Prevention (22) during the active phase of the disease (i.e., no longer experiencing symptoms, up to 14 days in mild cases). After this phase (median = 108 days after intake, IQR = 76–137), we asked these patients who were classified as experiencing mild COVID-19 (*n* = 993) about last-month substance use in three time-points: pre-COVID, just after COVID-19 acute phase (post-COVID acute phase) and in the period before survey (post-COVID follow-up phase).

### Sample

Residents of the municipality ≥18 years of age with suspected COVID-19 symptoms were encouraged to contact a specific website/phone platform for assessing COVID-19 (access at https://coronasaocaetano.org/) (baseline: April 6th to July 15th). They were invited to complete an initial screening questionnaire that included socio-demographic data; information on symptom type, onset, and duration; and recent contacts. People meeting the suspected COVID-19 case definition [i.e., having at least two of the following symptoms: fever, cough, sore throat, coryza, or change in/loss of smell (anosmia); or one of these symptoms plus at least two other symptoms consistent with COVID-19] were further evaluated, while people not meeting these criteria were advised to stay home and contact the service again were they to develop new symptoms or experience worsening of current ones (21). Patients were then asked to complete a risk assessment, of which there were no refusals. All patients were offered a home visit for self-collection of a nasopharyngeal swab (NPS – both nostrils and throat), which were collected at the patients' homes under trained healthcare supervision personnel. All pregnant women, and patients meeting pre-defined triage criteria for severe disease, were advised to attend a hospital service - either an emergency department or outpatient service, depending on availability. Additional details have been published elsewhere (21).

COVID-19 patients presenting with symptoms consistent with non-mild cases [i.e., dyspnea, tachypnea, persistent fever (≥72 h), altered level of consciousness, mental confusion], were evaluated in-person by a physician and were not included in the present cohort study (21). All the other patients who tested positive were classified as mild (21). and contacted over phone during the active COVID-19 phase (*N* = 1,983) were invited to participate in the present retrospective cohort study (online survey: September 14th to early October 27th). The response rate was 50.1%. We performed a comparison between those included in the present study (*N* = 993) and those who were not (*N* = 990), using logistic regression models. This comparison was performed to identify any potential baseline difference between the groups, which could generate bias to our outcome analysis (e.g., a higher number of COVID-19-related symptoms among those not included). Supplementary Table 1 presents a comparison between those that agreed to participate (*N* = 993) and those who did not (*N* = 990). We found that individuals aged 60 or greater were less likely to participate (OR = 1.99; 95%CI = 1.45–2.74). No significant differences were found regarding the total number of COVID-19 symptom(s). Our final analytical sample included 993 participants who completed the online survey.

### Measures

All COVID-19 measures were collected online via the dedicated Corona São Caetano web platform (access at https://coronasaocaetano.org/) or by phone. Substance use was assessed online only.

#### COVID-19 Symptoms

Patients testing positive for COVID-19 via RT-PCR were followed up to 14 days (a maximum of seven phone calls) from completing their initial questionnaire. They were contacted every 48 h by either a medical doctor or a medical student (supervised by a medical doctor) who completed another risk assessment and recorded any ongoing or new symptoms, following the COVID-19 clinical assessment protocol of São Caetano do Sul (21). All the COVID-19 symptoms listed by the CDC (22) were assessed during these contacts: fever or chills; cough; shortness of breath or difficulty breathing; fatigue; muscle or body aches; headache; new loss of taste or smell; sore throat; congestion or runny nose; nausea or vomiting; and diarrhea. The total number of CDC COVID-19 symptoms during the treatment was considered both as a continuous outcome (Aim 2) and exposure (Aims 3). In addition, each CDC COVID-19 symptom was also investigated as a categorical outcome for previous substance use (Aim 2).

#### Substance Use

We measured past-month use of alcohol, tobacco, cannabis, and non-medical use of benzodiazepines and analgesics (including opioid and non-opioid) using the ASSIST score for frequency of substance use (0 – none; 2 – monthly; 3 – fortnightly; 4 – weekly; 6 – daily or almost daily) (23). We assessed past-month substance use at three time points: the month prior to the disease diagnosis (pre-COVID); the month just after the active phase of the disease (post-COVID acute phase); and the last month before the survey (post-COVID follow-up phase). It is important to delineate the differences between post-COVID acute and post-COVID follow-up phases. The post-COVID follow-up phase allowed for variation among participants, depending on the time between the treatment intake and mental assessment. On average, the post-COVID follow-up phase assessment covered the period between 75 and 105 days after the treatment intake. In contrast, post-COVID acute phase assessment covered the month after the active phase of the disease, which could reach up to 44 days after the intake.

The psychometric properties of the Brazilian version of ASSIST proved to be satisfactory, supporting its use in patients of primary and secondary health care services (24). The Brazilian-version ASSIST scores for alcohol showed a good correlation with the AUDIT scores. This version also had good sensitivity and specificity in detecting alcohol, cannabis, and cocaine abuse and dependence, having the MINI-Plus diagnosis as the gold standard. Its reliability was good (Cronbach's alpha of 0.80 for alcohol, 0.79 for cannabis, and 0.81 for cocaine) (24). Shorter versions of ASSIST, including its frequency question, have been used to quickly screen substance use in clinical settings (25).

#### Potential Confounders

Lifetime diagnosis of psychiatric disorder (yes vs. no), age (categorical: 18–29; 30–39; 40–49; 50–59; and ≥60), sex (male vs. female), education (ordinal: no education; incomplete elementary education; complete elementary education; incomplete high school; complete high school; incomplete college; complete college), civil status (categorical: married; single; previously married; widow), income level (ordinal as defined by the Brazilian Institute of Geography and Statistics: no income; up to one times the typical salary for a minimum wage job; 1–3 times; 4–6; 6–9; 10–12; 13 or more), current health treatment for any acute or chronic medical condition (yes vs. no) and time between the treatment intake and mental assessment (continuous: median = 108, IQR = 76–137), were assessed as potential confounders.

### Statistical Analysis

STATA software version 16.2 was used to run the analysis. Initially, we conducted *t*-tests to compare pre-, post-COVID acute phase, and post-COVID follow-up phase substance use. We modeled the relationship between pre-COVID-19 substance use and the number of COVID-19 symptoms and using Poisson regression. We ran logistic regression models to quantify the association between pre-COVID substance use and each of the COVID-19 symptoms. Sensitivity analyses were conducted, excluding those with previous psychiatric disorders. Lastly, we ran ordinal regression modeling substance use at post-COVID acute phase and post-COVID follow-up phase time points, with number of COVID-19 symptoms and pre-COVID substance use as main exposures. All analyses were adjusted for potential confounders.

## Results

### Differences Between Pre- and Post-COVID-19 Substance Use

**Figure 1:**
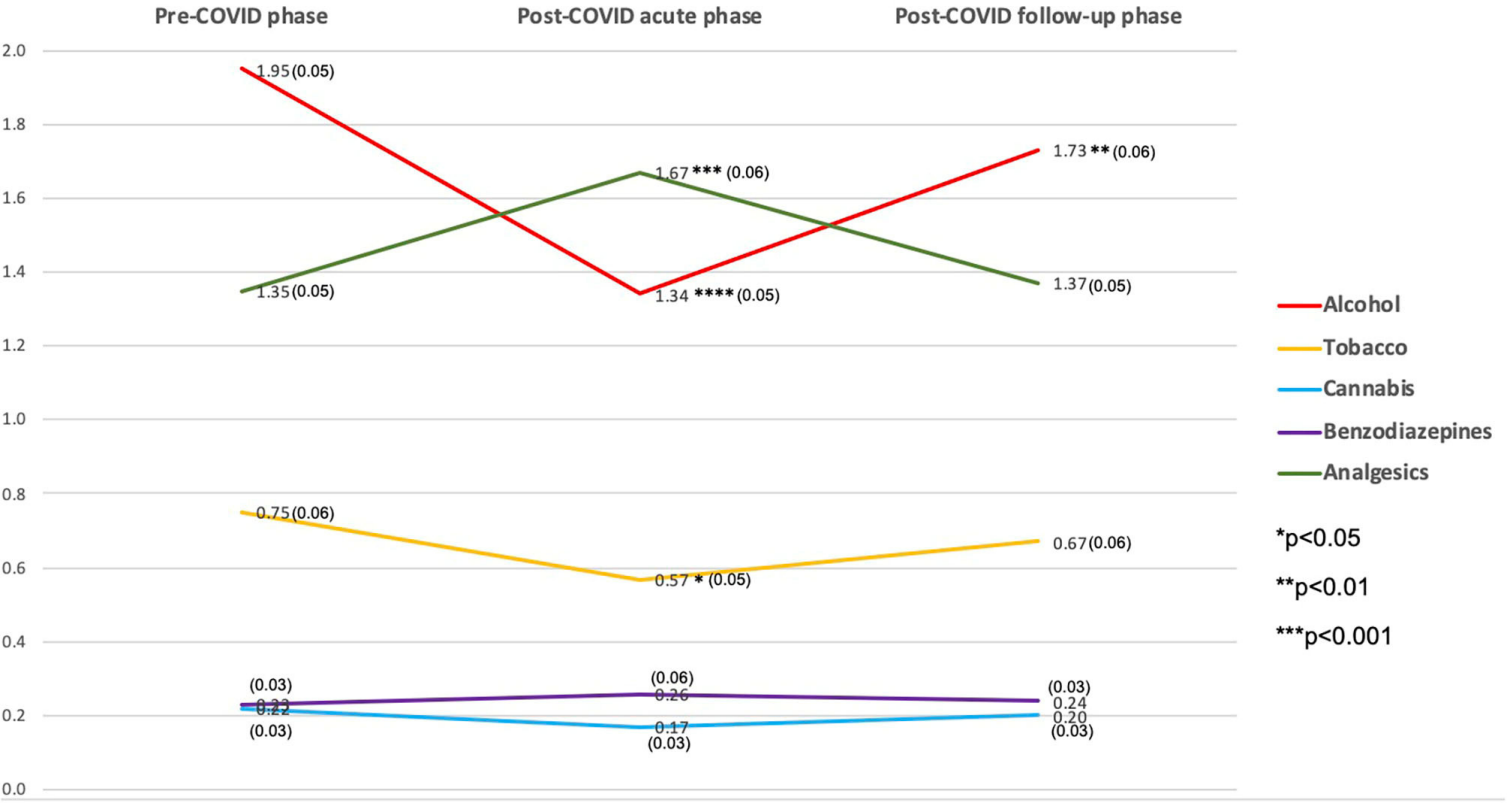
Last-month substance use frequency ASSIST score among 993 individuals who had mild COVID-19 in São Caetano do Sul, SP, Brazil, 2020 (y-axis = ASSIST frequency mean score).

Figure 1 and Supplementary Table 2 present past-month substance use for each substance across the three periods, along with the *t*-test results. Comparing substance use frequency scores, Alcohol had the highest ASSIST frequency scores in the pre-COVID and post-COVID follow-up periods, and analgesics in the post-COVID acute phase period. Post-COVID acute phase use was significantly lower for alcohol (1.34 vs. 1.95, *p* < 0.0001) and tobacco (0.57 vs. 0.75, *p* < 0.05); however, non-medical use of analgesics was higher (1.67 vs. 1.35, *p* < 0.001) compared to the pre-COVID period. Alcohol use was significantly lower in the post-COVID follow-up phase compared to the pre-COVID (1.73 vs. 1.95, *p* < 0.01). There were no significant changes for cannabis and non-medical benzodiazepine use throughout the period (Figure 1).

### Symptoms During the Active Phase of COVID-19

Table 1 presents the results of the multivariable Poisson regression model assessing the relationship between substance use and number of COVID-19 symptoms. There was no significant association between pre-COVID substance use and the number of COVID-19 symptoms. Table 2 presents the results of the multivariable logistic regression models assessing the impact of substance use for each of the symptoms of COVID-19. Alcohol use was positively associated with new loss of taste or smell (aOR = 1.09; 95%CI = 1.00–1.19) and congestion or runny nose (aOR = 1.09; 95%CI = 1.00–1.19). Non-medical benzodiazepine use increased the odds of experiencing shortness of breath or difficulty breathing by 53% (aOR = 1.53; 95%CI = 1.07–2.18), and was protective against experiencing a cough (aOR = 0.87; 95%CI = 0.71–0.98). There were no significant associations for pre-COVID tobacco, cannabis, and non-medical analgesic use. Sensitivity analyses (Supplementary Table 3) showed that, upon excluding those with previous psychiatric disorders or medical diseases, none of the associations found for previous alcohol or non-medical benzodiazepine use remained significant.

**Table 1:** Results of the Poisson regression model for number of CDC COVID-19 symptoms among 993 individuals who had mild COVID-19 in São Caetano do Sul, SP, Brazil.

**Outcome: CDC COVID-19 number of symptoms**	**Coef**.	**95%CI**	
**Exposure: pre-COVID phase ASSIST frequency score**			
Alcohol	0.007	−0.017	0.031
Tobacco	−0.003	−0.024	0.018
Cannabis	0.002	−0.038	0.043
Benzodiazepines	−0.005	−0.044	0.035
Analgesics	0.018	−0.006	0.042

*Adjusted for gender, age, city suburb, civil status, educational level, income, previous medical diseases, previous psychiatric disorder, time between intake and assessment*.

**Table 2:** Results of the logistic regression model for CDC COVID-19 symptoms among 993 individuals who had mild COVID-19 in São Caetano do Sul, SP, Brazil.

**Exposure: pre-COVID phase ASSIST frequency score**	**Alcohol**			**Tobacco**			**Cannabis**			**Benzodiazepines**			**Analgesics**		
	**aOR**	**95%CI**		**aOR**	**95%CI**		**aOR**	**95%CI**		**aOR**	**95%CI**		**aOR**	**95%CI**	
**Outcome: CDC COVID-19 symptoms**															
Fever or chills	0.97	0.84	1.13	1.04	0.92	1.18	0.97	0.73	1.27	1.05	0.80	1.37	0.92	0.87	1.19
Cough	0.97	0.89	1.06	0.97	0.90	1.05	1.01	0.87	1.17	0.84^*^	0.71	0.98	1.02	0.94	1.11
Shortness of breath or difficulty breathing	0.84	0.56	1.26	1.01	0.72	1.42	1.26	0.78	2.03	1.53^*^	1.07	2.18	0.96	0.71	1.31
Fatigue	1.00	0.92	1.10	1.02	0.95	1.11	1.02	0.88	1.18	1.07	0.92	1.24	1.03	0.95	1.13
Muscle or body aches	0.94	0.85	1.03	0.97	0.90	1.05	1.03	0.88	1.21	1.01	0.87	1.17	1.08	0.99	1.19
Headache	0.97	0.89	1.06	0.98	0.91	1.06	0.94	0.81	1.10	1.06	0.91	1.23	1.06	0.97	1.16
New loss of taste or smell	1.09^*^	1.00	1.19	0.97	0.90	1.04	0.91	0.79	1.05	0.98	0.84	1.14	1.04	0.95	1.13
Sore throat	0.95	0.86	1.06	1.01	0.91	1.11	1.04	0.87	1.23	1.03	0.86	1.24	0.96	0.86	1.08
Congestion or runny nose	1.09^*^	1.00	1.19	0.99	0.92	1.07	1.08	0.94	1.25	0.89	0.76	1.04	1.04	0.95	1.13
Nausea or vomiting	1.07	0.95	1.21	1.04	0.94	1.14	1.00	0.82	1.23	0.99	0.82	1.19	1.00	0.89	1.12
Diarrhea	1.03	0.90	1.17	0.93	0.82	1.06	0.90	0.68	1.19	1.01	0.80	1.27	0.95	0.85	1.12

*All the models adjusted for gender, age, city suburb, civil status, educational level, income, previous medical diseases, previous psychiatric disorder, time between intake and assessment*.

* *p < 0.05*.

### Post-COVID-19 Substance Use

Tables 3, 4 present the results of the ordinal regression models for post-COVID acute phase and post-COVID follow-up phase substance use, respectively. The number of COVID-19 symptoms was neither associated with post-COVID acute phase or post-COVID follow-up phase substance use. In general, those who used each substance tended to use this substance after the COVID-19 active phase. These associations had the highest coefficients in the ordinal regression models.

**Table 3:** Results of the ordinal regression model for post-COVID-19 acute phase substance use among 993 individuals who had mild COVID-19 in São Caetano do Sul, SP, Brazil.

**Outcome: post-COVID acute phase ASSIST frequency score**	**Alcohol**			**Tobacco**			**Cannabis**			**Non-medical benzodiazepine**			**Non-medical analgesic**		
	**Coef**.	**95%CI**		**Coef**.	**95%CI**		**Coef**.	**95%CI**		**Coef**.	**95%CI**		**Coef**.	**95%CI**	
**Exposures:**															
Number of CDC COVID-19 symptoms	−0.190	−0.450	0.069	−0.043	−0.199	0.112	−0.149	−0.389	0.091	0.035	−0.129	0.200	−0.053	−0.118	0.012
Pre-COVID-19 alcohol use	1.445^***^	1.296	1.593	−0.089	−0.321	0.143	0.025	−0.373	0.423	0.024	−0.239	0.287	0.138^**^	0.046	0.231
Pre-COVID-19 tobacco use	−0.002	−0.075	0.072	1.276^***^	1.100	1.452	0.047	−0.172	0.266	−0.075	−0.345	0.194	0.022	−0.056	0.100
Pre-COVID-19 cannabis use	0.020	−0.122	0.162	0.080	−0.140	0.299	1.730^***^	1.372	2.088	−0.648^*^	−1.169	−0.127	0.039	−0.121	0.199
Pre-COVID-19 non-medical benzodiazepines use	−0.100	−0.293	0.092	−0.005	−0.290	0.279	−0.611^*^	−1.147	−0.075	1.684^***^	1.380	1.989	0.179^*^	0.016	0.343
Pre-COVID-19 non-medical analgesics use	−0.111	−0.214	−0.007	−0.094	−0.296	0.109	0.314^*^	0.011	0.617	0.399^**^	0.159	0.639	1.413^***^	1.285	1.541

*All the models adjusted for gender, age, city suburb, civil status, educational level, income, previous medical diseases, previous psychiatric disorder, time between intake and assessment*.

* *p < 0.05;*

** *p < 0.01;*

*** *p < 0.001*.

**Table 4:** Results of the ordinal regression model for pot-COVID-19 follow-up phase substance use among 993 individuals who had mild COVID-19 in São Caetano do Sul, SP, Brazil.

**Outcome: post-COVID follow-up phase**	**Alcohol**			**Tobacco**			**Cannabis**			**Non-medical benzodiazepine**			**Non-medical analgesic**		
	**Coef**.	**95%CI**		**Coef**.	**95%CI**		**Coef**.	**95%CI**		**Coef**.	**95%CI**		**Coef**.	**95%CI**	
**Exposures:**															
Number of CDC COVID-19 symptoms	0.001	−0.069	0.072	−0.077	−0.259	0.106	−0.191	−0.450	0.069	0.150	0.002	0.298	0.054	−0.013	0.120
Pre-COVID-19 alcohol use	1.956^***^	1.783	2.128	0.129	−0.143	0.402	0.084	−0.319	0.487	−0.030	−0.269	0.209	0.099^*^	0.004	0.194
Pre-COVID-19 tobacco use	0.070	−0.005	0.144	1.613^***^	1.387	1.840	0.247^*^	0.038	0.456	0.053	−0.159	0.265	−0.036	−0.117	0.045
Pre-COVID-19 cannabis use	−0.044	−0.196	0.107	−0.082	−0.321	0.156	2.325^***^	1.862	2.788	−0.460^*^	−0.896	−0.023	0.120	−0.037	0.276
Pre-COVID-19 Non-medical benzodiazepines use	−0.101	−0.293	0.090	−0.121	−0.443	0.200	−0.908^**^	−1.540	−0.276	1.221^***^	0.990	1.452	0.167^*^	0.010	0.325
Pre-COVID-19 Non-medical analgesics use	0.042	−0.061	0.145	−0.064	−0.296	0.168	0.181	−0.146	0.508	0.315^**^	0.104	0.527	1.401^***^	1.274	1.528

*All the models adjusted for gender, age, city suburb, civil status, educational level, income, previous medical diseases, previous psychiatric disorder, time between intake and assessment*.

* *p < 0.05;*

** *p < 0.01;*

*** *p < 0.001*.

In addition to these strong associations found for each substance, we found some cross-substance effects throughout the COVID-19 active phase. Pre-COVID alcohol use was associated with non-medical analgesic use in post-COVID acute phase and post-COVID follow-up phases. Pre-COVID tobacco use was associated with post-COVID follow-up phase cannabis use. There was a positive bi-directional cross-substance association between non-medical benzodiazepine and analgesic use along the period evaluated in the study. An opposite situation was found for cannabis and non-medical benzodiazepine use, in which pre-COVID use of one substance was negatively associated with use of the other in the post-COVID acute phase and post-COVID follow-up phases.

## Discussion

The present study aimed to examine the pre- and post-infection frequency of substance use and their relationship with COVID-19 symptoms in mild patients. The number of COVID-19 symptoms was neither associated with pre- or post-infection substance use. Pre-infection alcohol and benzodiazepine use were associated with specific COVID-19 symptoms. Sensitivity analyses showed that such associations could be explained by people who use substances previous psychiatric and medical profile. Regarding variations in substance use, alcohol and tobacco use decreased, and non-medical analgesic use increased in the post-infection period. However, just the alcohol use remained lower in the post-COVID follow-up phase. Higher pre-COVID levels of tobacco and alcohol were associated with cannabis and non-medical analgesic and cannabis use in the post-COVID follow-up phase, respectively. Non-medical benzodiazepine use had negative and positive bi-directional associations with cannabis and non-medical analgesic use, respectively.

Previous studies investigated the COVID-19 vulnerability among patients with substance use disorders. An electronic health record study, which included data from more than 73 million patients, found that substance use disorder increased the risk of COVID-19 (12). They also found that individuals with substance use disorder had higher levels of pulmonary, kidney, cardiovascular, metabolic, liver, and immunological diseases, which increase the likelihood of experiencing more severe COVID-19-related outcomes (12). In the present study, we were not able to observe such a broad vulnerability. However, this study was restricted to participants with mild COVID symptoms. Notwithstanding that, our study found that the vulnerability to COVID-19 specific symptoms (e.g., shortness of breath or difficulty breathing, new loss of taste or smell, and congestion or runny nose) was not significant when excluding those with previous medical and psychiatric conditions. There are some possible explanations for the association of such specific symptoms with pre-COVID alcohol and benzodiazepine use. Alcohol Long-term alcohol use could have toxic effects on gustatory function (26) and can cause rhinosinusitis hyper-responsiveness, especially among those with previous clinical diseases (27). Non-medical and non-prescribed use of benzodiazepines has been largely correlated with anxiety disorders (28), which could explain the higher rates of dyspnea among this subpopulation during COVID-19.

Regarding substance use, mild COVID-19 patients may behave differently from the rest of the population who were not infected by the disease. There was a decrease in alcohol and tobacco use in the post-COVID acute phase in the present study, with the first remaining lower than the pre-COVID period in the post-COVID follow-up phase. No differences were found for cannabis use. These results contrast with the initial studies reporting increased substance use in the general population during the COVID-19 containment period in other countries, including the U.S. (15), U.K. (20), France (16), Belgium (17), but are more in line with the findings from Latin America and Caribbean (18) and Australia (13, 14). Decreased alcohol and tobacco use in mild COVID-19 patients seem to follow the decreased levels of substance use in individuals experiencing or being afraid of contracting diseases (29), rather than the increase found in those facing stressful situations (30). The increased non-medical analgesic use during the post-COVID acute phase could be explained by some popular reasons such as pain and tension relief (31), some of the symptoms experienced by a considerable number of patients in the post-COVID-19 period (32).

COVID-19 can increase the risk of some specific transitions among substances. The transitions from alcohol and tobacco to analgesics and cannabis, respectively, could be influenced by the disease-risk perception associated with these drugs. Alcohol and tobacco have been associated with several diseases, having a higher disease-risk perception (33, 34). On the other hand, cannabis and analgesics have a lower disease-risk perception, being associated with misperceptions of medical benefits (35, 36). In the acute post-COVID-19 phase, many patients experience very uncomfortable symptoms, such as fatigue, muscle weakness, pain, dyspnea, headache, and fever, which may impact functionality (7). The positive bi-directional association between benzodiazepines and analgesics is supported by many previous studies (37–39). However, others have found a negative association between the use of cannabis and benzodiazepines (40, 41), and have identified a substitutional role between them (42). These findings could explain the negative bi-directional relationship found in the present study.

The present study has several implications. Mild COVID-19 patients should be monitored for substance use in the post-infection period. Analgesic non-medical use preventive interventions should be implemented during the disease period. Focused preventive interventions increasing the perceived risk of cannabis use and non-medical use of benzodiazepines and analgesics among previous people who use substances could be of interest.

## Strengths and Limitations

A 50%-response rate is the main limitation of the present study. However, the patients included in the present study were just slightly different from those who did not attend the survey invitation. Despite the latter being older, no additional significant differences were found. In addition, we were able to collect data from a large clinical sample. The main issue for the generalization of our findings was the inclusion of individuals dependent on the public healthcare sector only. The use of an adapted measure of substance use could be cited as a limitation, but it was a feasible way of collecting timely data. Online data collection could be seen both as a strength and limitation. Undoubtedly, it allowed us to collect data quickly. However, online surveys assessing substance use are subject of two main types of biases: sampling and non-response bias (43). Online surveys could pose a challenge for achieving a high response rate among people who are less active online. In the present study, this could be the reason for a significantly lower response rate among older individuals. Thus, our findings are not generalizable to older adults. Unfortunately, we were also not able to assess whether social distance measures could have affected substance availability to our sample during the period of the study. However, São Paulo state adopted just a “partial lockdown” (i.e., industrial activities, construction, supermarkets, banks, pharmacies, pet-shops, health and basic services were allowed to remain open) during the period of the study (44). It is worth noting that drug supply did not seem to be affected even during periods of “full lockdown” (45).

## Conclusion

We could not replicate such a broad vulnerability to COVID-19 for people who use substances found in previous studies with samples with people with more severe COVID-19 and substance use disorders symptoms. Our study found that the vulnerability of people who use substance (i.e., alcohol and non-medical benzodiazepine) to COVID-19 specific symptoms disappeared when excluding those with previous medical and psychiatric conditions. Alcohol and tobacco use decreased and non-medical analgesic use increased in the post-COVID period. Only alcohol use remained lower in the post-COVID follow-up phase. Exposure to mild COVID-19 may predispose individuals increase non-medical analgesic use in the post-COVID period and should be the target of broad prevention interventions with mild COVID-19 patients. In addition, those who report previous substance use could be at-risk for a transition to cannabis use, non-medical use of benzodiazepines and analgesics, and could be the target of more focused preventive interventions. All mild COVID-19 patients should be monitored for substance use after the active phase of COVID-19.

## Data Availability Statement

The datasets presented in this article are not readily available because of confidential patient data. Requests to access the datasets should be directed to https://coronavirus.saocaetanodosul.sp.gov.br.

## Ethics Statement

The studies involving human participants were reviewed and approved by Comissão de Ética para Análise de Projeto de Pesquisa - CAPPesq. The patients/participants provided their written informed consent to participate in this study.

## Author Contributions

FI, TB, BZ, JG, VV, LBQ, JCSB, FEL, JT, AV, MEM, PG, SSM, and JMC-M designed the study. FI, JCSB, TB, BZ, and JMC-M wrote the protocol. FI, TB, BZ, JG, VV, LBQ, JCSB, and FEL managed data collection. TB, BZ, and JMC-M created the databank. JMC-M, JT, AV, MEM, PG, and SSM managed the literature searches and summaries of previous related work. JMC-M and SSM undertook the statistical analysis. FI, JT, AV, and JMC-M wrote the first draft of the manuscript. All authors contributed to and have approved the final manuscript.

## Conflict of Interest

The authors declare that the research was conducted in the absence of any commercial or financial relationships that could be construed as a potential conflict of interest.
