# Corrigendum: Substance Use in Mild-COVID-19 Patients: A Retrospective Study

**DOI:** 10.3389/fpubh.2021.703562

**Published:** 2021-06-16

**Authors:** Flavia Ismael, Beatriz Zaramella, Tatiane Battagin, João C. S. Bizario, Júlia Gallego, Victoria Villela, Lilian Bezerra de Queiroz, Fabio E. Leal, Julio Torales, Antonio Ventriglio, Megan E. Marziali, Priscila D. Gonçalves, Silvia S. Martins, João M. Castaldelli-Maia

**Affiliations:** ^1^Universidade Municipal de São Caetano do Sul, São Caetano do Sul, Brazil; ^2^ABC Center for Mental Health Studies, Santo André, Brazil; ^3^Faculdade de Medicina de Olinda, Olinda, Brazil; ^4^Department of Psychiatry, School of Medical Sciences, National University of Asunción, Asunción, Paraguay; ^5^Department of Clinical and Experimental Medicine, University of Foggia, Foggia, Italy; ^6^Department of Epidemiology, Mailman School of Public Health, Columbia University, New York, NY, United States

**Keywords:** COVID-19, alcohol, analgesics, cannabis, tobacco, benzodaizepine

In the original article, we neglected to include the funder **“NIDA”**, **“T32DA031099 (Hasin)”** to **“Priscila D. Gonçalves”**.

The authors apologize for this error and state that this does not change the scientific conclusions of the article in any way. The original article has been updated.

